# Supplementary material for: Analysis of ovarian transcriptomes reveals thousands of novel genes in the insect vector Rhodnius prolixus
Source: Sci Rep. 2021 Jan 21;11:1918. doi: 10.1038/s41598-021-81387-1 (PMC7820597; doi:10.1038/s41598-021-81387-1)
Supplement: Supplementary file 1 — Supplementary Figures. [file 41598_2021_81387_MOESM1_ESM.pdf]

Supplementary figures

Stage-specific ovarian transcriptomes reveals thousands of novel genes in the insect vector *Rhodnius prolixus*

Vitor Lima Coelho[1], Tarcísio Fontenele de Brito[1], Ingrid Alexandre de Abreu Brito[1], Maira Arruda Cardoso[1], Mateus Antonio Berni[1], Helena Maria Marcolla Araujo[1,3], Michael Sammeth[2] and Attilio Pane[1]*

* Corresponding author: apane@icb.ufrj.br

[1] Institute of Biomedical Sciences (ICB), Federal University of Rio de Janeiro, Rio de Janeiro, Brazil.

[2] Institute of Biophysics Carlos Chagas Filho (IBCFF), Federal University of Rio de Janeiro, Rio de Janeiro, Brazil.

[3] Instituto Nacional de Ciência e Tecnologia em Entomologia Molecular (INCT-EM), Rio de Janeiro, Brazil.

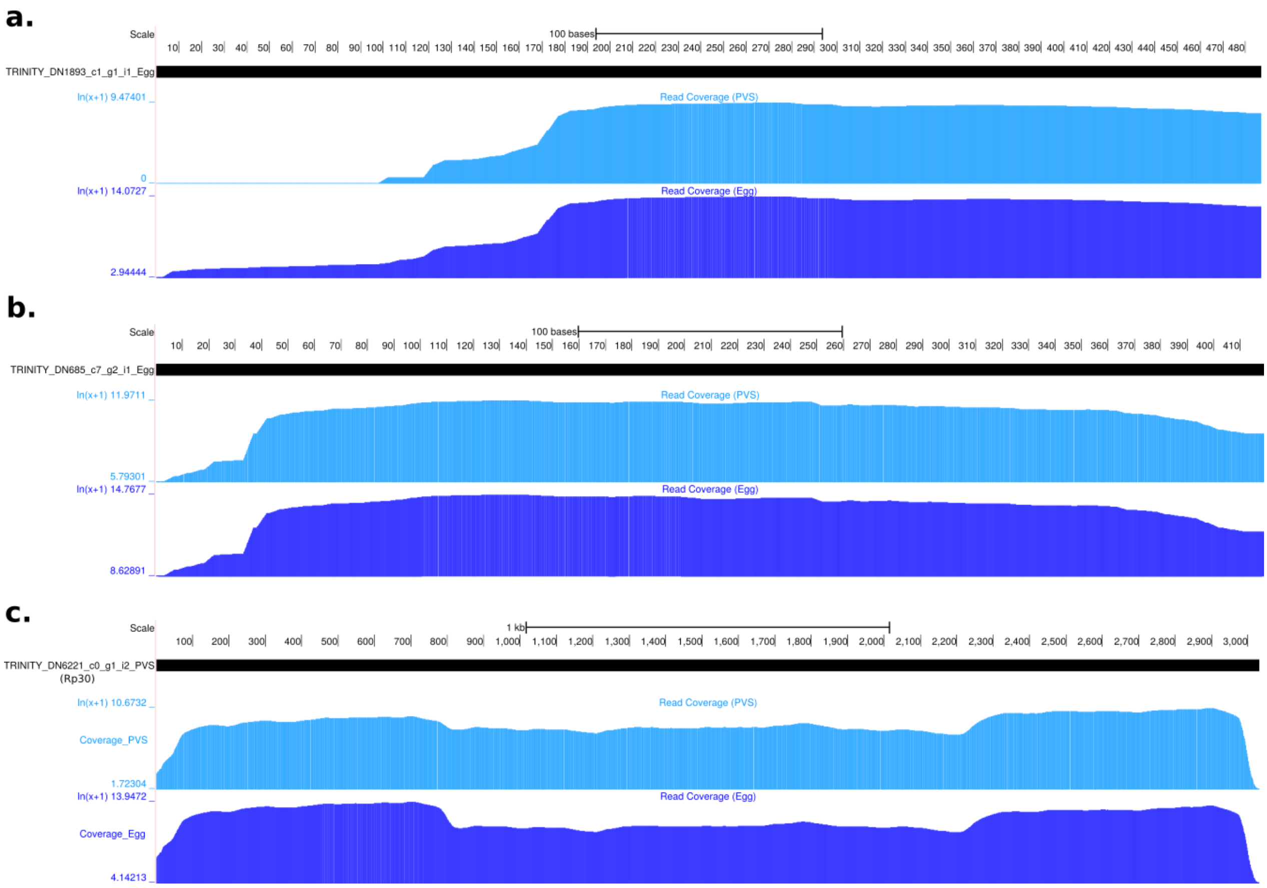

Figure S1. Most-expressed putative protein-coding contigs. Three contigs that were accountable for more than 80% of the unmapped reads assembled by the *de novo* transcriptome assembly approach. Coverage is calculated by the number of mapped reads normalized by natural logarithm.

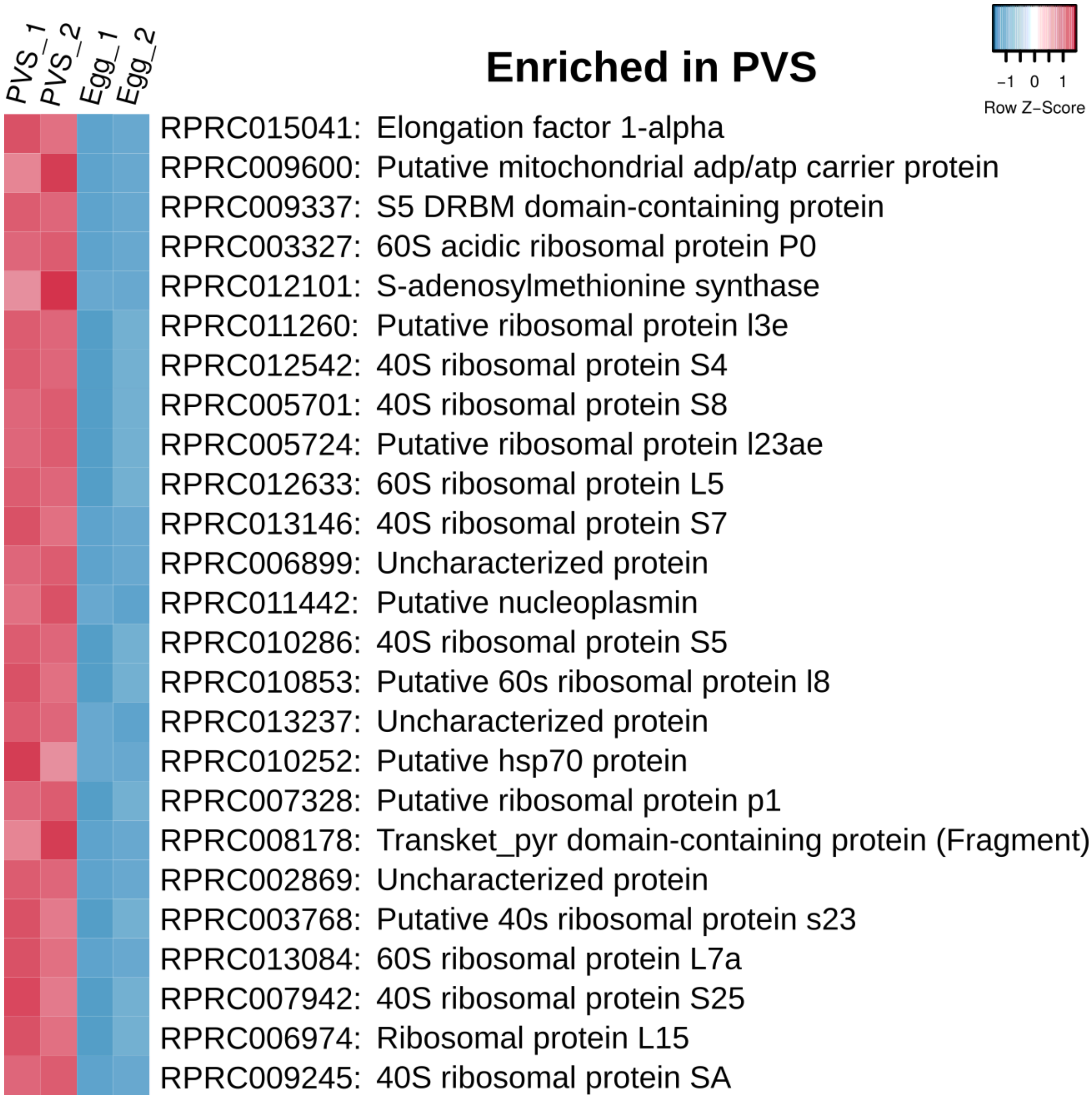

Figure S2. Top 25 differentially most-expressed genes of previtellogenic stage (PVS). Genes are ordered by average normalized expression (CPM). Putative protein products were assigned according to the Uniprot database.

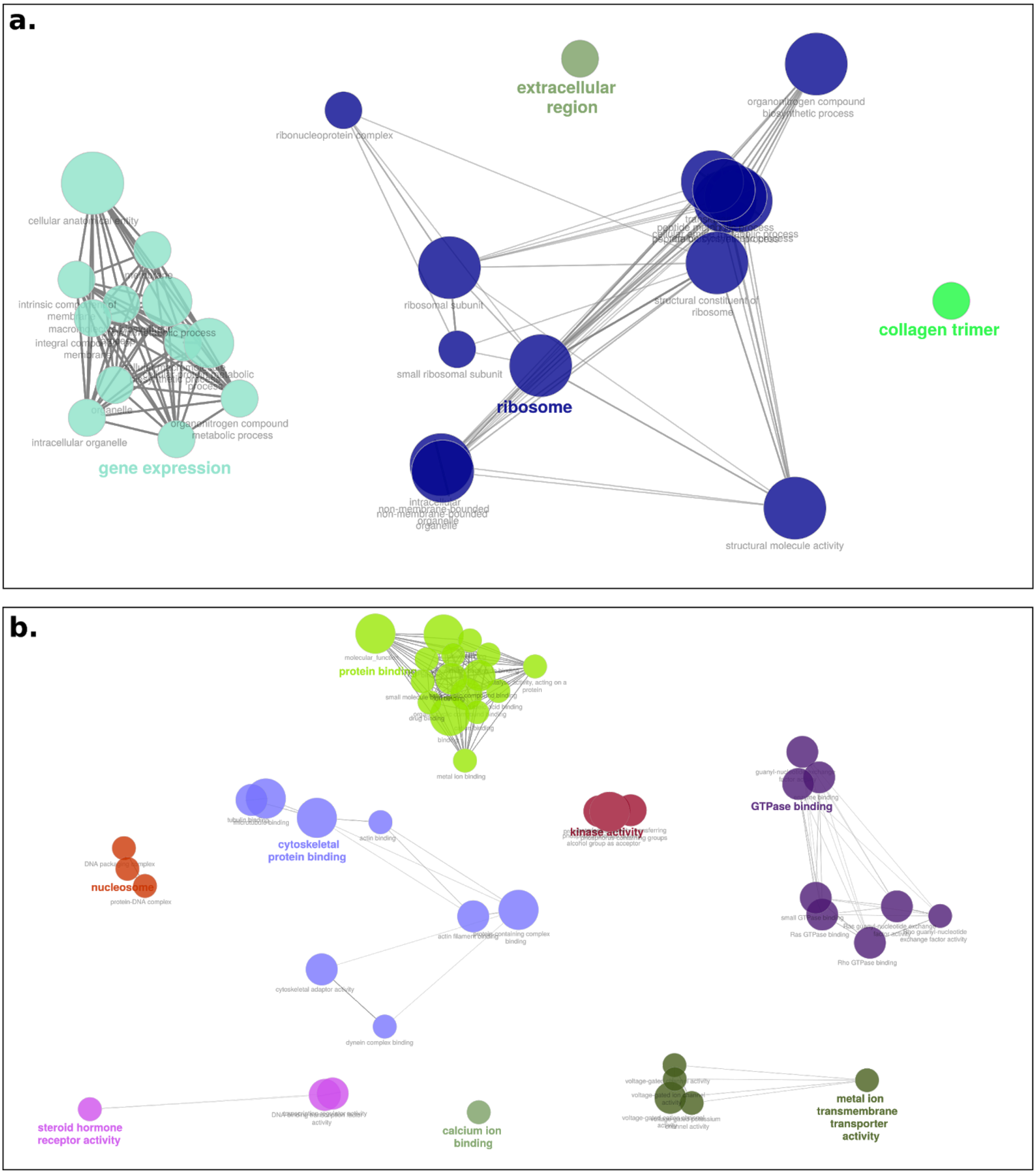

Figure S3. Functional networks of the gene ontology enrichment analysis. (A) Functionally grouped network of the genes enriched in PVS, i. e., down-regulated genes (B) Functionally grouped network of the genes enriched in Egg, i. e., up-regulated genes. Networks are represented using a prefuse force directed layout.
